# Two Kinds of Health

**Published:** 1920-11-13

**Authors:** 


### THE PUBLIC WELL-BEING.

### Two Kinds of Health.

It is not our province in these columns to discuss political questions or to intervene in the relationships of capital and labour. Such delicate matters of debate are best left where they belong. But there is one point of view from which it appears legitimate to make reference to the important Congress which has just taken place on the subject of co-partnership in industry. In his address to the Congress Lord Emmott, who is well qualified to speak, pointed out that co-operation of this kind is the ideal which many employers and captains of industry are now presenting again. They are seeking, he added, to dissipate that antagonism between capital and labour which is too often falsely represented as "negligible and unending," and which threatens at times to turn all industrial operations into a permanent condition of economic warfare. These observations come at a time when a large number of responsible men on the side both of capital and labour have been expressing themselves in similar terms. It is evident that men are more and more seriously turning their attention to the task of finding some means of establishing peace and contentment in industry; and it is evident that this co-partnership principle, in spite of its recent and occasional setbacks, will soon become practical politics. What, perhaps, a good many even of its most earnest advocates have not yet realised is the profound effect which the realisation of their ideal will have not only upon economic conditions, but upon the physical health of the community. There is perhaps scarcely a medical man in the country with a large general practice who has failed to perceive the connection between industrial contentment and physical well-being, or who would not have to report in recent times an increase in the number of patients suffering from various forms of nerve strain who come to him for relief. There is, in fact, a very definite relationship between the two things. Economic health, all things being equal, means physical health; and on that ground alone this steadily growing movement which the word "co-partnership" connotes is to be welcomed by everybody interested in the advancement of our race. Moreover, the setting up of any method of industrial control which gives all classes of the workers an incentive to work and a live interest in their labour, and thus produces contentment at the same time that it increases output, will also in the long run add immeasurably to the national wealth and so make possible the expenditure of a portion of that wealth on the many schemes urgently in request for improving the nation's health. There is hardly a hospital in England to-day which is not seriously in need of funds. There is no health work, either in public administration or in the realm of research, which is not limited on every hand by the overwhelming sense of financial stringency. It is, therefore, no far-fetched argument to say that peace in industry is an essential part of the health problem.

